# Sex Difference in Triathlon Performance

**DOI:** 10.3389/fphys.2019.00973

**Published:** 2019-07-24

**Authors:** Romuald Lepers

**Affiliations:** CAPS UMR1093, UFR STAPS, Faculté des Sciences du Sport, Institut National de la Santé et de la Recherche Médicale (INSERM), Université de Bourgogne-Franche Comté, Dijon, France

**Keywords:** gender difference, swimming, cycling, running, Ironman, human physiology

## Abstract

This brief review investigates how sex influences triathlon performance. Performance time for both Olympic distance and Ironman distance triathlons, and physiological considerations are discussed for both elite and non-elite male and female triathletes. The relative participation of female athletes in triathlon has increased over the last three decades, and currently represents 25–40% of the total field. Overall, the sex difference in both Olympic and Ironman distance triathlon performance has narrowed across the years. Sex difference differed with exercise mode and exercise duration. For non-elite Ironman triathletes, the sex difference in swimming time (≈12%) is lower than that which was evidenced for cycling (≈15%) and running (≈18%). For elite triathletes, sex difference in running performance is greater for Olympic triathlon (≈14%) than it is for Ironman distance triathlon (≈7%). Elite Ironman female triathletes have reduced the gap to their male counterparts to less than 10% for the marathon. The sex difference in triathlon performance is likely to be due to physiological (e.g., VO_2max_) and morphological (e.g., % body fat) factors but hormonal, psychological and societal (e.g., lower participation rate) differences should also be considered. Future studies should address the limited evidence relating sex difference in physiological characteristics such as lactate threshold, exercise economy or peak fat oxidation.

## Introduction

Triathlon is a unique endurance sport that combines swimming, cycling and running over a variety of distances (Bentley et al., 2002). Top male triathletes can nowadays finish an Olympic distance triathlon (1.5 km swim, 40 km cycle, and 10 km run) in less than 2 h and an Ironman (3.8 km swim, 180 km cycle, and 42 km run) distance triathlon in less than 8 h and 30 min (Rüst et al., 2013; Gallmann et al., 2014).

The number of females who compete in triathlon has increased since the 1990s but the rate of participation in triathlon events remains lower for females compared to males, with the female rate varying between 25 and 40% of the total field (Lepers, 2008; Lepers et al., 2013b; Wonerow et al., 2017). Nowadays, female triathletes have similar opportunities to train and compete than males in most parts of the world, but female participation rates remain lower than those of males, and particularly so in the case of long distance triathlons.

Females have progressively reduced the gap between their triathlon performance and that of their male counterparts over time (Lepers, 2008; Rüst et al., 2012b, 2013; Lepers et al., 2013a; Meili et al., 2013; Gallmann et al., 2014). The number of female triathletes who finish an Ironman distance triathlon in under 9 h has increased yearly, from 1 in 1991 to 23 in 2017^1^. In 2018, the Swiss woman Daniela Ryf won the Ironman Triathlon World Championship for the fourth time in a row, in a time of 8 h and 26 min, and placed 25th overall. She reduced the gap to the male winner (who finished the race in 7 h and 52 min) to 7.1% of total time^2^ which is much less than the 10–12% sex difference that is typically evidenced by elite endurance athletes (Joyner, 2017).

Although the sex difference in endurance performance has received considerable research attention, most studies have focused on running (Joyner, 2017). Previous investigations have, however, pointed out some male-female differences in selected physiological, biomechanical, nutritional, training and medical aspects of triathlon performance (O’Toole and Douglas, 1995; Hausswirth and Lehénaff, 2001; Laursen and Rhodes, 2001; Jeukendrup et al., 2005; Hausswirth and Brisswalter, 2008; Vleck et al., 2014). This brief review investigates how sex influences triathlon performance. Performance time for Olympic distance and Ironman distance triathlons, and physiological characteristics between female and male triathletes are discussed.

## Sex Difference in Performance

The sex differences in triathlon performance have been described for both elite and non-elite triathletes but more data are available for Ironman distance than for sprint or Olympic distance triathlons (Lepers, 2008; Lepers and Maffiuletti, 2011; Rüst et al., 2012a, b, 2013; Etter et al., 2013; Lepers et al., 2013a, b; Gallmann et al., 2014; Wonerow et al., 2017). The sex difference in total triathlon performance has decreased over the last three decades and currently varies between 12 and 18%, depending on both event distance and athlete ability level (Lepers et al., 2013a). With the exception of the Ironman distance triathlon World Championship, which takes place in Hawaii every year, the other World triathlon Championships take place in different towns, with different course topographies, and climatic conditions, making year-to-year absolute comparisons difficult. In Hawaii, the climatic conditions could also differ, affecting the performances. Despite these limitations, it still possible to compare males and females in terms of relative performances. Moreover, in contrast to Ironman triathlons, the international level Olympic distance triathlons have all been draft legal since 1997. It is consequently difficult to compare sex differences in overall performance between Olympic and Ironman distance triathlons.

### Sex Difference in Ironman Triathlon Performance

The Ironman distance triathlon World Championships, which have been held in Kona (HI, United States) since 1982 with only a few changes in the course, have been frequently used as a model to analyze the sex difference in Ironman performance (Lepers, 2008; Lepers and Maffiuletti, 2011; Rüst et al., 2012b; Lepers et al., 2013b).

By analyzing the Hawaii Ironman triathlon performances of the top 10 non-elite triathletes from the age groups between 18 and 64 years old, Lepers and Maffiuletti (2011) showed that the sex differences in 3.8 km swim, 180 km cycle, and 42 km run and total times were 12, 15, 18, and 16%, respectively. Independent of age, the sex difference in swimming time was lower than that which was evidenced for cycling and running. The sex difference in cycling time was lower than that for running. The lower sex difference in swimming performance may be explained by higher economy and mechanical efficiency of swimming in females compared to males (Zamparo et al., 2005). However, the lower sex difference in cycling performance as compared to running performance remains difficult to explain. In the literature, the sex difference in performance is usually lower in elite athletes than it is in non-elite athletes (Joyner, 2017). A confounding factor in non-elite athletes is age; indeed some studies have pooled data from different age groups when they have analyzed sex differences in performance of non-elite athletes (Lepers and Maffiuletti, 2011; Lepers et al., 2013a).

For elite athletes (i.e., top 10 male and female finishers), the sex difference in total performance has decreased over the last few decades, from 15% in the 1990s to 11% in 2012 (Lepers, 2008; Rüst et al., 2012b). During the period from 1983–2012, the sex differences in performance remained relatively stable at around 12.5% for swimming and cycling, whilst the sex difference in running performance decreased from 13.5 to 8% (Rüst et al., 2012b). The reasons for a greater improvement in females’ running performance at the Ironman during the 1983–2012 period are not clear because both males and females had the opportunities to use new training methods. One explanation could be a sex difference in race strategy. Females, using a more constant pacing strategy and not a fast-starting pacing strategy during the bike, may save energy for the marathon running. However, given the contrast previous findings (Wu et al., 2014; Angehrn et al., 2016), this hypothesis needs to be confirmed with further studies examining Ironman pacing strategy for both males and females.

In 2018, the weather conditions at the Ironman World Championship were favorable, with a light tail wind during the second part of the bike course. It was less hot than usual during the marathon. Both the elite male and elite female course records were broken (see footnote 2). Table 1 shows the sex differences in the Ironman World Championship records in 2018. The female Ironman record holder, thanks to a strong bike performance, was only 4% slower on the bike than the male record holder, lowering the sex difference in total Ironman performance to 7.1% between sexes. However, the performances of both male and female winners are not representative of the rest of the elite field. In contrast to Daniela Ryf, who performed relatively poorly in the swim and relatively well on the bike, the top 10 female cyclists remained 10.9% slower on the bike section than the top 10 male cyclists (Table 1). In addition, the sex difference in marathon performance of the top 10 runners (6.6%) appears lower than the sex difference in performance for the top 10 swimmers (9.1%) and cyclists (10.9%). These observations suggest that nowadays elite female Ironman triathletes are able to reduce the gap with their males counterparts to less than 10% of total performance, thanks to improvement in their marathon running performance. Nevertheless, it is clear that training to improve relative weaknesses in bike performance should be a major consideration for females aiming to maximize overall performance and to minimize the gap to their male counterparts.

**TABLE 1:** Split times and corresponding sex differences at the 2018 Ironman Triathlon World Championship (Hawaii) for the winners and the top 10 performers in each discipline and overall.

**Hawaii Ironman triathlon (2018)**		**Swim 3.8 km**	**Cycle 180 km**	**Run 42 km**	**Total**
Male winner – Patrick Lange		50:37	4:16:05	2:41:32	7:52:39
Female winner – Daniela Ryf		57:26	4:26:07	2:57:05	8:26:16
Sex difference (%)		13.5	3.9	9.6	7.1
Top 10 males	Mean	48:21	4:13:28	2:50:55	8:04:02
	SD	00:49	02:27	04:55	05:57
Top 10 females	Mean	52:47	4:41:10	3:02:08	8:48:05
	SD	01:56	05:54	04:28	10:44
Sex difference (%)	Mean	9.1	10.9	6.6	9.1
	SD	3.1	1.6	1.0	0.9

*Publicly available data obtained from the Ironman Corporation’s Web site at http://eu.ironman.com/triathlon/events/americas/ironman/world-championship/results.aspx#axzz5hwN1WJ9o.*

### Sex Difference in Olympic Distance Triathlon Performance

The sex difference in Olympic distance triathlon performances has received relatively less attention than that of the Ironman format in the research literature (Etter et al., 2013; Meili et al., 2013; Wonerow et al., 2017). The sex difference in non-elite Olympic distance triathlon performance has been examined by Etter et al. (2013) at national level, and by Wonerow et al. (2017) at international level. Etter et al. (2013) analyzed the sex differences in the non-drafting Zurich (Switzerland) triathlon, for the top 5 athletes overall, within each 5-year age group between the ages of 20 and 60 years, over the period 2000–2010. The sex difference in 1.5 km swimming, 40 km cycling, 10 km running and total event times was 18.5, 15.5, 18.5, and 17.1%, respectively. Sex difference differed with exercise mode, independent of athlete age. Indeed, in this study the sex difference in performance appeared to be significantly lower for cycling than it was for swimming and running, but the reasons for this finding were unclear. Sex difference in Olympic distance triathlon has also been examined at World Championship level, during the period 2009–2014, by Wonerow et al. (2017). Unfortunately, the marked variability of results both over the 5-year period of study and across age groups did not allow for conclusions to be drawn about a possible effect of locomotion mode on the sex differences in performance. The changes in race conditions across events and the confounding effect of age make difficult to clearly elucidate the sex differences in non-elite triathlon performance.

As elite-level international Olympic distance triathlons have all been draft legal for several years, the pacing over the course, especially during the bike section, could influence the sex difference in performance (Vleck et al., 2008). Only one study has examined the sex difference in triathlon performance for elite triathletes during draft legal races (Le Meur et al., 2009). Le Meur et al. (2009) showed that both female and male elite triathletes adopt similar positive pacing strategies during the swim and run legs of draft legal races. However, compared to females males pushed the pace harder during the swim-to-cycle transition with high levels of cycling power output at the beginning of the bike session, and female triathletes were more affected by changes in slope during the triathlon cycle and run. By analyzing the performance of elite male and female triathletes in international Olympic distance triathlons from 2009 to 2012, Rüst et al. (2012b) found that the sex difference in running (14.3%) was greater than that which was evidenced for swimming (9.1%) and cycling (9.5%). In light of this observation, the relatively lower gender difference in cycling versus running may be associated with drafting, pacing and/or cadence on the bike (Bernard et al., 2003; Le Meur et al., 2009). However, the relative effects of these factors on the cycling and subsequent running performance of males as compared to females remain to be fully explored, and certainly warrant further study.

### Comparison Between Olympic Distance and Ironman Distance Triathlons

**FIGURE 1:**
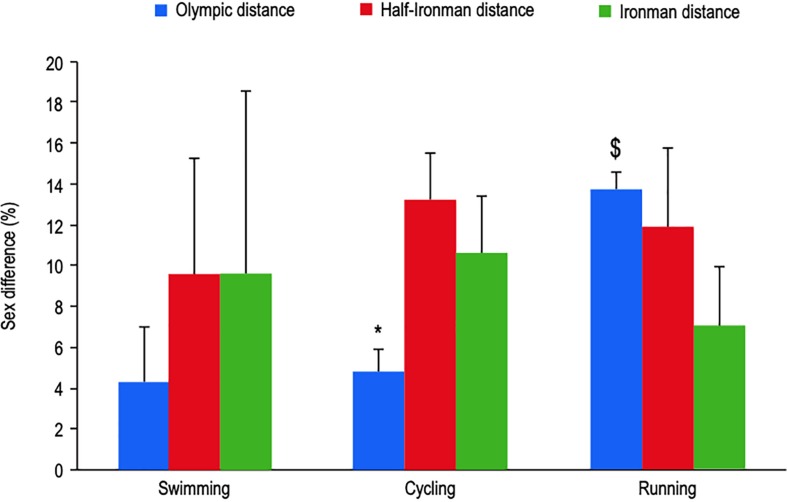
Sex difference in time for swimming, cycling and running between the top 10 females and males at three triathlons considered as the World Championship of the distance in 2018. Olympic distance : World Triathlon Series – Grand Final (Gold Coast, Australia), Half-Ironman distance : Half-Ironman Triathlon Word Championship (Port Elizabeth, South Africa), Ironman distance : Ironman Triathlon World Championship (HI, United States). ^*^different from Half-Ironman distance, ^$^different from Ironman distance; *p* < 0.05.

Figure 1 shows the sex difference in time for swimming, cycling and running at three 2018 World Championships of the different distances. Whilst not yet established as statistically significant, sex difference in swimming performance appears to be lower for Olympic distance as compared to half- and full- Ironman distance performance. The lower sex difference in cycling performance that has been observed for Olympic distance versus half-Ironman distance events is difficult to explain and requires further investigation. It could be due to different racing strategies between males and females and/or a greater drafting benefit for females as compared to males within Olympic distance races. In contrast, sex difference in running performance appears to be greater for Olympic triathlon than it is for Ironman distance triathlon. The sex difference in running time clearly decreased with running distance from 13.7% for Olympic distance to 7.0% for Ironman distance. Similar observations were made by Lepers and Stapley (2010). Such an increase in the sex difference in running performance with a decrease in running distance is intriguing. Several hypotheses can be proposed for why this might occur. Firstly, the difference may be explained by the drafting conditions of Olympic distance triathlon. It has been shown that drafting can improve subsequent running performance (Hausswirth et al., 2001) and that fast runners likely benefit most from drafting during the triathlon cycle (Hausswirth et al., 1999). Differences between the sexes in drafting strategy can influence running performance. Male triathletes may also benefit more from drafting than females because they tend to ride in larger packs (Landers et al., 2008). The exercise duration of both the running course and the event overall could also explain the lower sex difference in Ironman marathon performance as compared to 10 km running. Given this point, and the physiological implications of differing distances (and intensities) of event, there are a number of physiological and morphological factors, which may underpin performance differences between of genders and, therefore, warrant further discussion.

## Physiological Considerations

Sex difference in triathlon performance may be explained in part by morphological and physiological factors. Triathlon performance is related to body morphology (Ackland et al., 1998; Landers et al., 2000). Elite female athletes generally have 7–12% more body fat than males (Fleck, 1983; Heydenreich et al., 2017). As fat is buoyant in water, women are less penalized than men in swimming than they are within terrestrial events such as cycling and running. Male triathletes also possess a larger muscle mass, greater muscular strength and lower relative body fat than female triathletes (Knechtle et al., 2010a). It has been shown that both male and female triathletes’ morphology has evolved since the 1990s. This may possibly be a result of changes in race distances and race tactics (Landers et al., 2013). It is worth noting that low body fat was associated with faster race times in male Ironman non-elite triathletes but not in females (Landers et al., 2000; Knechtle et al., 2010b).

The physiological characteristics of both elite and non-elite triathletes, particularly those which are considered to be the determinants of endurance performance [i.e., maximal oxygen uptake (VO_2max_), anaerobic/ventilatory threshold and economy of motion] have been extensively studied (Millet et al., 2011). However, limited research has examined the physiological characteristics for both male and female triathletes within the same study. One of the first studies that reported physiological data for both sexes was that of O’Toole et al. (1987). For male and female Ironman triathletes, respectively, the latter authors recorded treadmill VO_2max_ values of 68.8 vs. 65.9 ml kg^–1^ min^–1^, and cycle VO_2max_ values of 66.7 vs. 61.6 ml kg^–1^ min^–1^. O’Toole et al. (1989) also reported VO_2max_ values for groups of triathletes during treadmill running to range from 52.4 to 72 ml kg^–1^ min^–1^ in males and from 58.7 to 65.9 ml kg^–1^ min^–1^ in females, respectively. The sex difference in VO_2max_ may depend on the level of the triathletes, the way that it is measured (i.e., whether this is done during running or cycling) or event distance specialization (e.g., between those who are focusing their training on Olympic distance vs. Ironman distance events) (Millet et al., 2011). Nevertheless, it would appear that elite females exhibit relatively lower VO_2max_ values than males, irrespective of exercise mode. Furthermore, it would seem that the discrepancy between bike and run VO_2max_ is greater for females than it is for males. This may reflect a relatively greater weakness in the bike discipline in female triathletes and a need for this to be prioritized in their development.

Bunc et al. (1996) examined the physiological profile of top 17 year old male and female triathletes and found that VO_2max_ was 20% lower for the females than it was for the males (56.1 vs. 67.9 ml kg^–1^ min^–1^). However, in both cases, the athletes′ ventilatory threshold corresponded to 82% of VO_2max_. Similarly, Millet and Bentley (2004) found that for elite junior and senior triathletes, the sex difference in VO_2max_ was equal to 22% (74 vs. to 61 ml kg^–1^ min^–1^ for males and females, respectively). The ventilatory threshold was similar in senior males and females and corresponded to 74–77% of VO_2max_.

These findings corroborate data for endurance running athletes for whom both absolute and relative VO_2max_ is lower for female athletes than it is for male athletes. Sex difference in VO_2max_ still persists when it is expressed per kilogram of fat free mass (Joyner, 2017). These differences are attributed to a combination of higher body fat in females and lower red cell mass and hemoglobin for a given body weight (Schmidt and Prommer, 2010).

Whilst VO_2max_ is lower for female triathletes than it is for male triathletes, lactate threshold (the exercise intensity associated with a marked rise in blood lactate and expressed as a percentage of VO_2max_) appears to be similar in both sexes when measured in cycling and running (Millet et al., 2011). A determinant of a high lactate threshold is the ability of the mitochondria in muscles to increase in volume in response to training (Holloszy and Coyle, 1984). An increase in the capillary density in muscles also plays a role in how long athletes might be able to sustain exercise at high intensities (Coyle et al., 1988). There is no evidence that these adaptive capacities are less in females than in males. Of the three main determinants of triathlon performance, less is known about running economy and cycling efficiency than VO_2max_ or lactate threshold. Running economy or energy cost can be defined as oxygen uptake during running at a certain speed. Energy cost is higher during the running part of a triathlon than it is during an isolated run (Hausswirth et al., 2000). The study of Millet and Bentley (2004) suggested that there was no difference between males and females in running economy. However, in the aforementioned study the absolute test speed was lower for females than it was for males. Thus, the oxygen cost relative to a certain reference speed (e.g., 15 km/h) was markedly different between sexes and represented a factor in the performance difference. Data on sex difference in cycling efficiency for triathletes is missing. Hopker et al. (2010) have examined the cycling efficiency in male and female competitive cyclists. These authors found that gross efficiency was higher in female cyclists for submaximal intensities (150 W and 180 W). Males also exhibited a higher oxygen cost of “unloaded” cycling, suggesting that in addition to work rate, leg volume/mass may be an important determinant of the observed differences in cycling efficiency between male and female well-trained cyclists. These results for competitive cyclists need to be confirmed in well-trained triathletes. In conclusion, it appears that the main physiological difference between females and males that affects triathlon performance is VO_2max_. Lactate threshold and locomotion economy probably do not explain much of the sex difference in triathlon performance but these two factors require further investigations in male and female elite triathletes.

Other factors such as sex differences in thermoregulation and fat oxidation could conceivably play a role. Some studies suggested that females, because of their smaller body size, can better tolerate hot and humid racing conditions than males (Dennis and Noakes, 1999; Marino et al., 2000). Lighter runners produce and store less heat at the same running speed, thus females may be less susceptible than males to overheating during a long race in oppressive weather. These data leads to the prediction that females might compete against males most successfully in long distance triathlons where overheating is particularly common, such as the Hawaii Ironman triathlon. Another interesting sex difference is the fact that females appear to oxidize proportionately more lipid and less carbohydrate during endurance exercise. Indeed, studies investigating sex differences in the metabolic response to prolonged submaximal exercise have found that females have a lower respiratory exchange ratio, and attenuated muscle glycogen utilization as compared to males (Tarnopolsky et al., 1990; Carter et al., 2001). This could offer females the possibility that, in triathlons that take several hours to complete, their supply of liver and muscle glycogen will outlast that of men. However, Vest et al. (2018) found that peak fat oxidation did not predict Ironman race time independently of aerobic capacity in females. On the other hand, Frandsen et al. (2017) found that maximal fat oxidation rate exerted an independent influence on Ironman performance in males. These contradictory results suggest that additional research is required to better understand the role of fat oxidation in both male and female triathlon performance.

It has been shown that females can sometimes finish ultra-marathons in times similar to those of the males who can beat them in marathons. Similarly, when males and females with equivalent marathon times are pitted against each other in ultra-marathons, the females tend to win (Speechly et al., 1996; Bam et al., 1997). It would be interesting to verify if this finding also applies to triathlon performance, i.e., when males and females have equivalent short distance triathlon time performances, do females tend to win over the Ironman distance and longer events such as ultra-triathlon events (Knechtle et al., 2011)? We could also ask what females could do to mitigate the gap to the males if they drop down to shorter events- where they are at more of a disadvantage relative to them.

## Conclusion and Perspectives

The sex difference in triathlon performance, representing a difference of approximately 10–20% of total time, depends upon the disciplines, the distances and the level of competitors. The sex difference in performance is likely to be due to physiological and morphological factors but hormonal, psychological and societal differences (such as lower participation rates) should also be considered. There is a growing pool of females getting involved in the sport earlier/younger, and consequently training specifically for triathlon for a greater period. This will probably filter into the top level. Recently, elite female triathletes have reduced the gap to their male counterparts during the marathon section of Ironman distance triathlon running, with the current difference standing at less than 10%. The reasons why such running improvements in females are not observed for short-distance triathlon remains not clear. It seems that cycling is the discipline with the most potential for improved female triathlon performance, especially at Ironman distance. Future studies should address the limited evidence relating sex difference in some physiological characteristics of triathletes such as lactate threshold, exercise economy or peak fat oxidation. It is important to better understand the sex difference in triathlon performance, both to promote more female participation and to help female triathletes to achieve their maximal performance.

## Author Contributions

The author drafted the manuscript.

## Conflict of Interest Statement

The author declares that the research was conducted in the absence of any commercial or financial relationships that could be construed as a potential conflict of interest.

## Footnotes

1 https://www.tri247.com/triathlon-features/analysis/sub-9-hour-iron-women-triathletes

2 http://eu.ironman.com
